# Distinct Phenotypes of Human Prostate Cancer Cells Associate with Different Adaptation to Hypoxia and Pro-Inflammatory Gene Expression

**DOI:** 10.1371/journal.pone.0096250

**Published:** 2014-05-06

**Authors:** Linda Ravenna, Lorenzo Principessa, Alessandra Verdina, Luisa Salvatori, Matteo Antonio Russo, Elisa Petrangeli

**Affiliations:** 1 CNR, Institute of Molecular Biology and Pathology, Rome, Italy; 2 Department for the Development of Therapeutic Programs, CRS, Regina Elena Cancer Institute, Rome, Italy; 3 Department of Sensory Organs, “Sapienza” University of Rome, Rome, Italy; 4 Department of Experimental Medicine, “Sapienza” University of Rome, Rome, Italy; 5 Department of Cellular and Molecular Pathology, IRCCS San Raffaele Pisana, Rome, Italy; Southern Illinois University School of Medicine, United States of America

## Abstract

Hypoxia and inflammation are strictly interconnected both concurring to prostate cancer progression. Numerous reports highlight the role of tumor cells in the synthesis of pro-inflammatory molecules and show that hypoxia can modulate a number of these genes contributing substantially to the increase of cancer aggressiveness. However, little is known about the importance of the tumor phenotype in this process. The present study explores how different features, including differentiation and aggressiveness, of prostate tumor cell lines impact on the hypoxic remodeling of pro-inflammatory gene expression and malignancy. We performed our studies on three cell lines with increasing metastatic potential: the well differentiated androgen-dependent LNCaP and the less differentiated and androgen-independent DU145 and PC3. We analyzed the effect that hypoxic treatment has on modulating pro-inflammatory gene expression and evaluated the role HIF isoforms and NF-kB play in sustaining this process. DU145 and PC3 cells evidenced a higher normoxic expression and a more complete hypoxic induction of pro-inflammatory molecules compared to the well differentiated LNCaP cell line. The role of HIF1α and NF-kB, the master regulators of hypoxia and inflammation respectively, in sustaining the hypoxic pro-inflammatory phenotype was different according to cell type. NF-kB was observed to play a main role in DU145 and PC3 cells in which treatment with the NF-kB inhibitor parthenolide was able to counteract both the hypoxic pro-inflammatory shift and HIF1α activation but not in LNCaP cells. Our data highlight that tumor prostate cell phenotype contributes at a different degree and with different mechanisms to the hypoxic pro-inflammatory gene expression related to tumor progression.

## Introduction

Prostate cancer exhibits a heterogeneous cell population including rare cancer stem cells (CSC) and pluripotent progenitors (Ps) embedded in a mass of cell types at various degrees of differentiation. The relative abundance of CSC+Ps and the differentiation of bulk cells correlate with tumor malignancy [1], [2]. However, few data are available on the impact of the phenotype of bulk tumor cells in adapting to environmental stress and particularly to hypoxia. Hypoxia is a reduction in the normal level of tissue oxygen tension which may occur in human pathologies. Recent studies have shown that hypoxia promotes a more aggressive metastatic phenotype in human cancers such as breast [3], glioblastoma [4], thyroid [5], colon [6], pancreatic [7] and in particular prostate tumors [8]–[10] which is associated with a poor prognosis. Hypoxia inducible factors (HIFs) are key regulators of the transcriptional response to hypoxic stress [11]. They are heterodimers formed by an O_2_ sensitive α subunit and a constitutively expressed β subunit (HIF1β). Three inducible isoforms of HIFα are present in mammals. HIF1α and HIF2α are the best characterized and structurally similar isoforms [12]. HIF3α is the more distantly related one with numerous splice variants [13]. In the presence of oxygen, HIF1α undergoes proteasomal degradation. Under hypoxic conditions, it accumulates in the cell nuclei, forms heterodimers with HIF1β and binds hypoxia response elements at target gene loci. Also HIF2α and HIF3α present hypoxic stabilization and binding to HIF1β although with different kinetics. Both HIF2α and HIF3α appear expressed in a cell-specific manner and play non redundant roles in adapting to hypoxia and in hypoxic tumor growth and progression [14], [15]. Increasing evidence indicates that the inflammatory microenvironment is a further contributing factor leading to cancer development in the prostate [16], [17]. Inflammatory gene response depends on several transcription factors, among which NF-kB plays a central role. The classical form of NF-kB is the heterodimer p50/p65. Following activation, NF-kB dimers translocate into the nucleus where they can undergo phosphorylation, bind target genes and stimulate transcription [18]. A cross-talk between the NF-kB and the HIF pathways has been documented extensively [19]–[21]. Indeed, the NF-kB subunits p50 and p65 directly interact with the NF-kB consensus site on the HIF1α promoter and contribute to basal levels of HIF1α mRNA and protein in some models [22], [23]. On the other hand, hypoxia seems to activate NF-kB dependent gene transcription [24]. However, the underlying mechanisms linking hypoxia to inflammation and inflammation to tumor progression still remain elusive. Recent reports have highlighted the role of hypoxic tumor cells in the synthesis of inflammatory-related molecules in breast [3], glioblastoma [4], thyroid [25] and prostate [26] malignant cancer progression. In addition, they demonstrated that a coordinated pathway including inflammatory and reparative molecules is present in tumor tissue in the absence of detectable leukocyte infiltrate (CD45+) and is up-regulated in transformed cells.

The present study was carried out in order to analyze the relative importance of the HIF and NF-kB pathways in the modulation of the hypoxic inflammatory gene expression in prostate cell models showing distinct phenotypes with increasing differentiation. Clarifying the specific involvement of these two pathways in intratumor heterogeneous cells could have useful fall-out on clinical research and therapy [27]. To this end, we performed our experiments on the well differentiated, androgen-dependent LNCaP and on the less differentiated, androgen-independent DU145 and PC3 tumor prostate cell lines with low, moderate and high metastatic potential, respectively [28]–[32]. We took into consideration a representative set of genes related to the innate immune response greatly involved in prostate tumor progression that were shown upregulated in tumor tissue [26], [33]. These include: the damage receptor for advanced glycation end products (RAGE) and the purine receptor (P2X7R), the vascular epidermal growth factor A (VEGF) involved in tumor angiogenesis, the inducible enzymes ciclo-oxygenase-2 (COX2) responsible for prostaglandins synthesis, the acute phase protein pentraxin 3 (PTX3) and the C-X-C chemokine receptor 4 (CXCR4) of stromal cell-derived factor, regulator of invasive growth and metastasis formation. Moreover, we analyzed the levels of heme-oxygenase-1 (HO1), the rate limiting enzyme in heme degradation, as a prototype of anti-inflammatory regulator [34], [35]. To evaluate the impact of NF-kB in the hypoxia driven modulation of the analyzed pro-inflammatory genes, we studied the effects of the NF-kB inhibitor parthenolide. The contribution of HIF1α and the combined action of NF-kB and HIF1α were analyzed in the androgen-independent DU145 cells stably knockdown for HIF1α.

## Materials and Methods

### Cell lines and cell culture

Human prostate cancer cell lines LNCaP, DU145 and PC3 were obtained from the American Type Culture Collection. LNCaP were grown in RPM1-1640 medium, DU145 and PC3 in D-MEM medium (Invitrogen) supplemented with 10% v/v inactivated fetal bovine serum (Thermo Scientific HyClone) and 100 U/ml penicillin+100 µg/ml streptomycin. Puromycin dihydrocloride (Sigma-Aldrich) 2 µg/ml was always added to the medium of HIF1α knockdown clones. Cells were maintained under standard normoxic conditions (95% air and 5% CO_2_) in a humidified incubator at 37°C. For experiments in hypoxic conditions, cells were seeded in dishes in complete growth medium at a density depending on the length of treatment to reach subconfluence when the analysis was performed. On the day of experiment, the medium was replaced with preconditioned hypoxic medium and cell cultures were subjected to hypoxia in a sealed modular incubator chamber (Billups-Rothenberg) flushed with 1% O_2_, 5% CO_2_ and 94% N_2_ according to the manufacturer's instructions and cultured at 37°C. Parthenolide (Sigma-Aldrich) treatments were carried out at a concentration of 5 µM for the time indicated, always preceded by a 2 h pre-treatment in normoxia.

### Protein extraction and western blot assay

Nuclear extracts were prepared by a nuclear extract kit (Active Motif). A total of 5–20 µg proteins were resolved on Tris-HCl polyacrylamide gels and electrophoretically transferred to polyvinylidene difluoride membranes (Invitrogen). Membranes were blocked in PBS-fat free milk 5% (Bio-Rad Laboratories) for 1 h, probed with the appropriate primary antibody overnight at 4°C and subsequently incubated with peroxidase conjugated secondary antibodies for 1 h at room temperature. Immunocomplexes were visualized using an enhanced chemiluminescent kit (EuroClone). Digital images of the resulting bands were quantified by the Quantity One software package (Bio-Rad Laboratories) and expressed as arbitrary densitometric units.

The following primary and secondary antibodies were used: mouse anti-HIF1α (1∶500, BD Bioscience); mouse anti-HIF2α and rabbit anti-p50 (1∶500, 1∶1000, Novus Biologicals); rabbit anti-HIF3α (1∶1000, Aviva Biological Systems); mouse anti-p65 (1∶2000, Santa Cruz Biotechnology); rabbit anti phospho-NF-kB p65 (Ser 276) (1∶1000, Cell Signaling); mouse anti β-actin (1∶10000, Sigma Aldrich); horseradish peroxidase conjugated anti-mouse and anti-rabbit (1∶2000, Bio-Rad Laboratories).

### RNA isolation and real-time quantitative polymerase chain reaction

Total RNA was extracted by Trizol reagent and reverse transcribed using Superscript II Reverse Transcriptase and random primers (Invitrogen) according to the manufacturer's instructions. Quantitative RT-PCR was done with the ABI Prism 7000 Sequence Detection System (Life Technologies). TaqMan Gene Expression Assay kits were used for HIF1α, HIF2α, HIF3α, VEGF, RAGE, P2X7R, COX2, PTX3, CXCR4, HO1 and 18S rRNA (Life Technologies) with the manufacturer's default cycling conditions. The mRNA level of each gene was quantified using a suitable standard curve and normalized to the housekeeping gene 18S rRNA.

### Stable gene silencing

Mission shRNA Bacterial Glycerol Stock harboring sequence verified shRNA lentivirus plasmid vectors (clone numbers TRCN0000003810 and TCRN0000010819) which express short hairpin RNA (shRNA) targeting HIF1α (HIF1α shRNA) and the non-targeting shRNA negative control plasmids (NTshRNA) were purchased from Sigma-Aldrich. Bacterial cultures were amplified and the shRNA plasmids purified (PureLink, Invitrogen) and transfected into DU145 cells. Stable transfections were performed using Lipofectamine™2000 (Invitrogen) according to the manufacturer's instructions. Briefly, 10^6^ cells were seeded in dishes with 6 cm diameter. The following day, cells were transfected with 4 µg plasmid in a medium without antibiotics. Six h later, the medium was replaced with a standard medium and, 24 h after starting transfection, cells were trypsinized and seeded at different dilutions. After another 24 h, the selective antibiotic puromycin hydrochloride was added at the final concentration of 2 µg/ml. Ten selected colonies for each plasmid vector were amplified and tested for HIF1α mRNA expression. Colonies with a reduced HIF1α mRNA level (∼20% of average wt value) for silencing plasmid vectors and a mRNA level comparable to wt cells for NTshRNA plasmid vector were checked for HIF1α nuclear protein by western blot, after 4 h hypoxic stimulation. To reduce the variability of hypoxic response, we performed our experiments on three different knockdown clones and we present the mean ± SE of the results obtained from each clone. For control, a mix of three non targeting plasmid vectors transfected clones was used. NTshRNA DU145 and wt cells did not show any significant differences in the normoxic and hypoxic levels of all the parameters analyzed. Therefore, we indicate as “wt” the mean ± SE of the data obtained both from wt and NTshRNA DU145 cells.

### Statistical analysis

Each experiment was performed at least three times and representative results are shown. Values in bar graphs are given as the means ± SE. Statistical significance for single comparisons of normally distributed data was determined by Student's t test or by Mann-Whitney rank sum test for data not normally distributed. All statistics were analyzed by PRISM program. P-values less than 0.05 were considered significant (*/°/# P<0.05, **/°°/## P<0.01,***/°°°/### P<0.001).

## Results

### Protein nuclear translocation and mRNA transcription of HIF1α HIF2α and HIF3α in hypoxic prostate cancer cells LNCaP, DU145 and PC3

**Figure 1:**
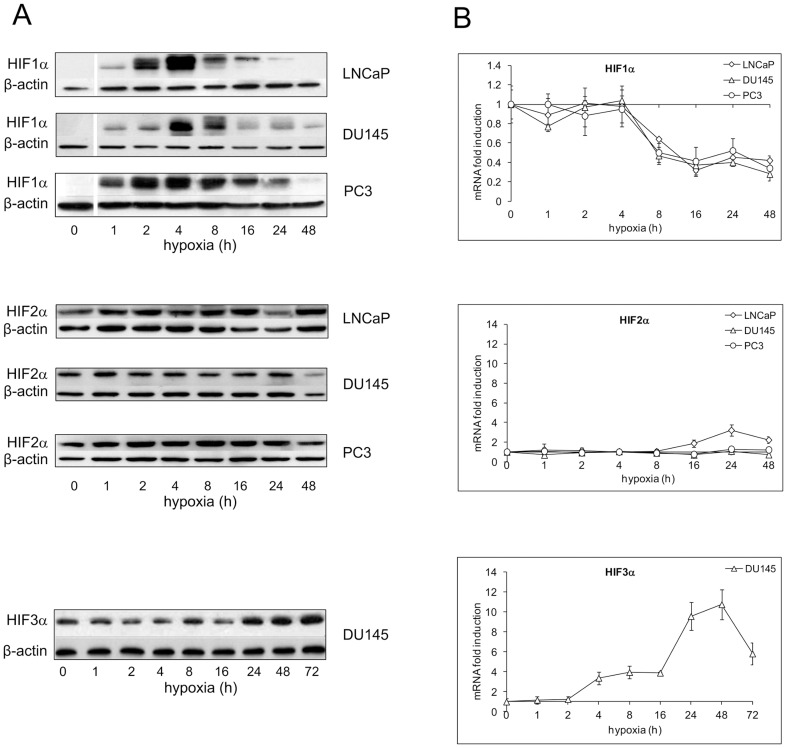
Time-course of hypoxia on HIF1α, HIF2α and HIF3α protein nuclear level and mRNA expression in LNCaP, DU145 and PC3 cells. Cell were exposed to 1% O_2_ from 1 up to 48–72 h, according to the experimental design or left untreated. **A** Protein expression was detected in nuclear extracts by immunoblot analysis. A representative experiment for each gene in each studied cell lines is shown. β-actin was used as a loading control. **B** mRNA determinations were performed by real-time PCR, normalized to the housekeeping gene 18S rRNA and expressed as fold induction with respect to normoxic controls (set at 1). Mean values ± SE.

Nuclear translocation is a measure of the activation of HIF isoforms. We therefore characterized the nuclear expression profile of HIF1α, HIF2α and HIF3α in the tumor prostate cell lines LNCaP, DU145 and PC3 following oxygen deprivation (Figure 1A). In normoxic control, HIF1α protein was undetectable or present at a very low level. One % oxygen increased its nuclear translocation in all the examined cell lines with similar kinetics. Nuclear accumulation started early after oxygen deprivation (1 h), reached a peak after 4 h and declined close to the base level by 48 h. HIF2α was present in the nucleus in all the cell models also in normoxia and its quantity did not appear significantly modulated in any of the studied models. A 72 kDa nuclear HIF3α protein was detectable only in normoxic DU145 cells where a late induction was also observed starting after 24 h stimulation. Densitometric analysis evidenced a maximum increase of 2.8±0.8 folds compared to normoxic cells after 48 h oxygen withdrawal and a slow decline to values still above those of controls after 72 h hypoxia (data not shown).

The effects of hypoxia on HIF1α, HIF2α and HIF3α mRNA levels in the studied cell models are depicted in Figure 1B. HIF1α mRNA was expressed in all unstimulated cells. In the hypoxic environment, HIF1α mRNA level remained unchanged for 4 h and then abruptly decreased to 40-50% of the base value and stayed stably low till 48 h treatment. Also HIF2α mRNA was expressed in all normoxic cell lines and showed a late and slight increase only in the androgen-dependent LNCaP after 24 and 48 h oxygen deprivation. HIF3α mRNA was detectable only in DU145. In this cell model, hypoxia determined mRNA up-regulation that preceded the increase in nuclear protein, starting after 4 h and reaching a peak after 48 h stimulation (10.8 ± 1.5 fold induction).

Altogether, our data highlight that the HIF1**α** isoform, out of the three analyzed, is the main player in the response to hypoxia regardless of cell phenotype. HIF3**α** activation is cell specific and does not appear to be related to cancer cell aggressiveness.

### Hypoxia activates the NF-kB pathway in DU145 and PC3 but not in the androgen-dependent LNCaP cells

**Figure 2:**
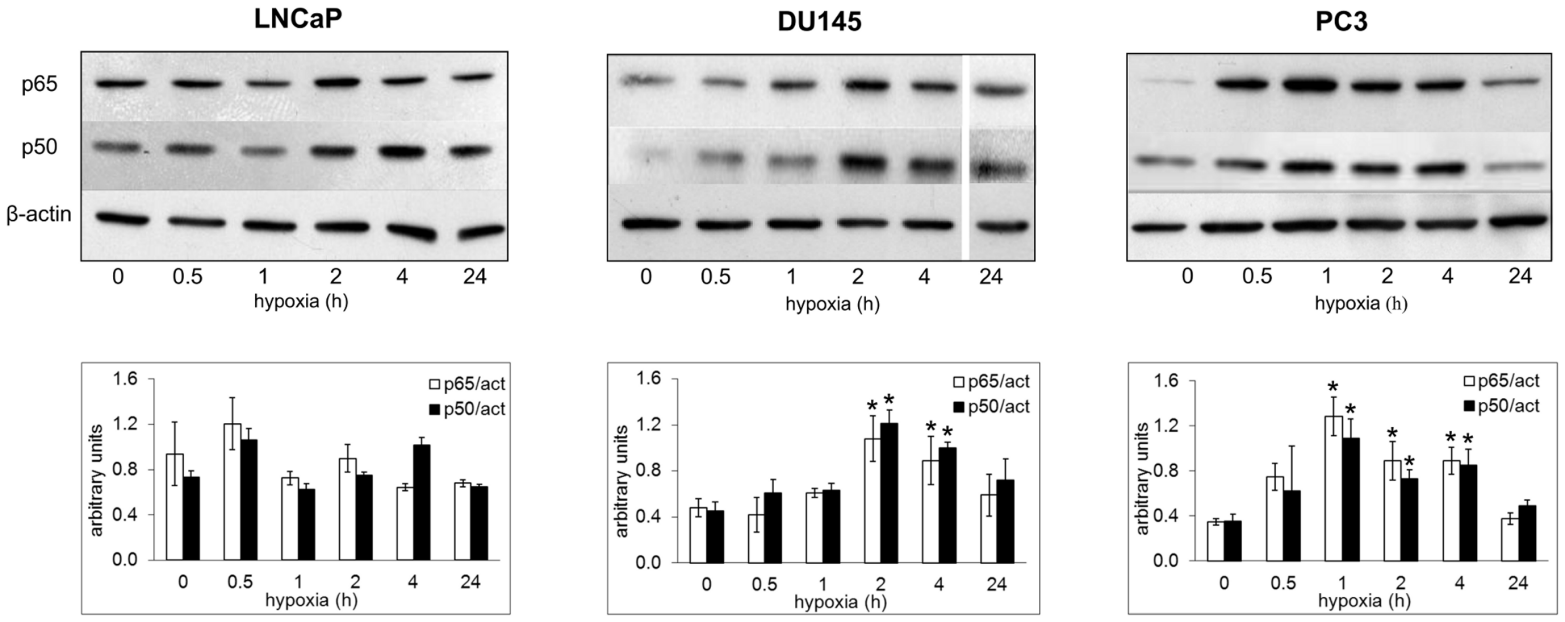
Hypoxic regulation of NF-kB nuclear expression in LNCaP, DU145 and PC3 cells. Cell were exposed to 1% O_2_ from 0.5 up to 24 h or left untreated. After the times indicated, cells were processed and the nuclear content of p50 and p65 was detected by immunoblot analysis. β-actin was used as a loading control. A representative experiment for each gene in all the studied cell lines is shown. Mean densitometry of NF-kB p50 and p65 relative to β-actin ± SE is also evidenced and expressed as arbitrary units. * Hypoxic cells *vs* normoxic control cells.

We evaluated the effect of hypoxia on the NF-kB status by western blot analysis, quantifying the amount of nuclear p50 and p65 subunits in time-course experiments of oxygen deprivation. Basal NF-kB activation was observed in all cell lines. Nuclear translocation of both p50 and p65 were significantly increased compared to normoxic cells in PC3 (1–4 h) and DU145 (2–4 h), while oxygen deprivation failed to significantly modulate nuclear NF-kB level in LNCaP cells within the same time span (Figure 2).

These results provide evidence that hypoxia has a different impact on NF-kB pathway according to tumor cell phenotype.

### Hypoxic upregulation of the inflammatory-related phenotype in prostate tumor cells

We then studied the role of hypoxia in modulating the synthesis of pro-inflammatory molecules in the described prostate tumor cell lines. To this end, we selected a number of representative members of the gene families involved in inflammation and in tissue repair overexpressed in prostate tumor or metastasis and their expression in time-course of acute (2 and 4 h) and chronic (24, 48, 72 h) hypoxic stimulation was measured by real time PCR.

Qualitative and quantitative differences were observed in the base level and in the hypoxic induction of the selected molecules according to cell phenotype and metastatic potential.

LNCaP cells were the less active producers of the molecules studied under normoxia. We did not detect transcripts for PTX3 and COX2 and all the other genes, except for CXCR4, were significantly less transcribed compared to androgen-independent DU145 and PC3 cells (Table 1).

**Table 1:** Normoxic expression levels of pro-inflammatory genes in LNCaP, DU145 and PC3 cells.

	VEGF	RAGE	P2X7R	PTX3	COX2	CXCR4	HO1
**LNCaP**	0.10±0.02	0.13±0.02	0.20±0.02	not detected	not detected	0.80±0.04	0.03±0.01
**DU145**	***0.61±0.05	***3.22±0.43	not detected	*** 22.53±3.67	***20.62±2.35	*2.84±0.29	***0.92±0.13
**PC3**	***0.35±0.04	***0.69±0.09	***1.52±0.2	*** 249±54	***1515±356	0.70±0.09	**0.26±0.04

Normoxic expression levels of pro-inflammatory genes in LNCaP, DU145 and PC3 cells. Values express the mRNA level of each gene quantified by real-time PCR and normalized to the housekeeping gene 18S rRNA. Mean ± SE. Asterisks indicate a significant higher value compared to LNCaP cells.

**Figure 3:**
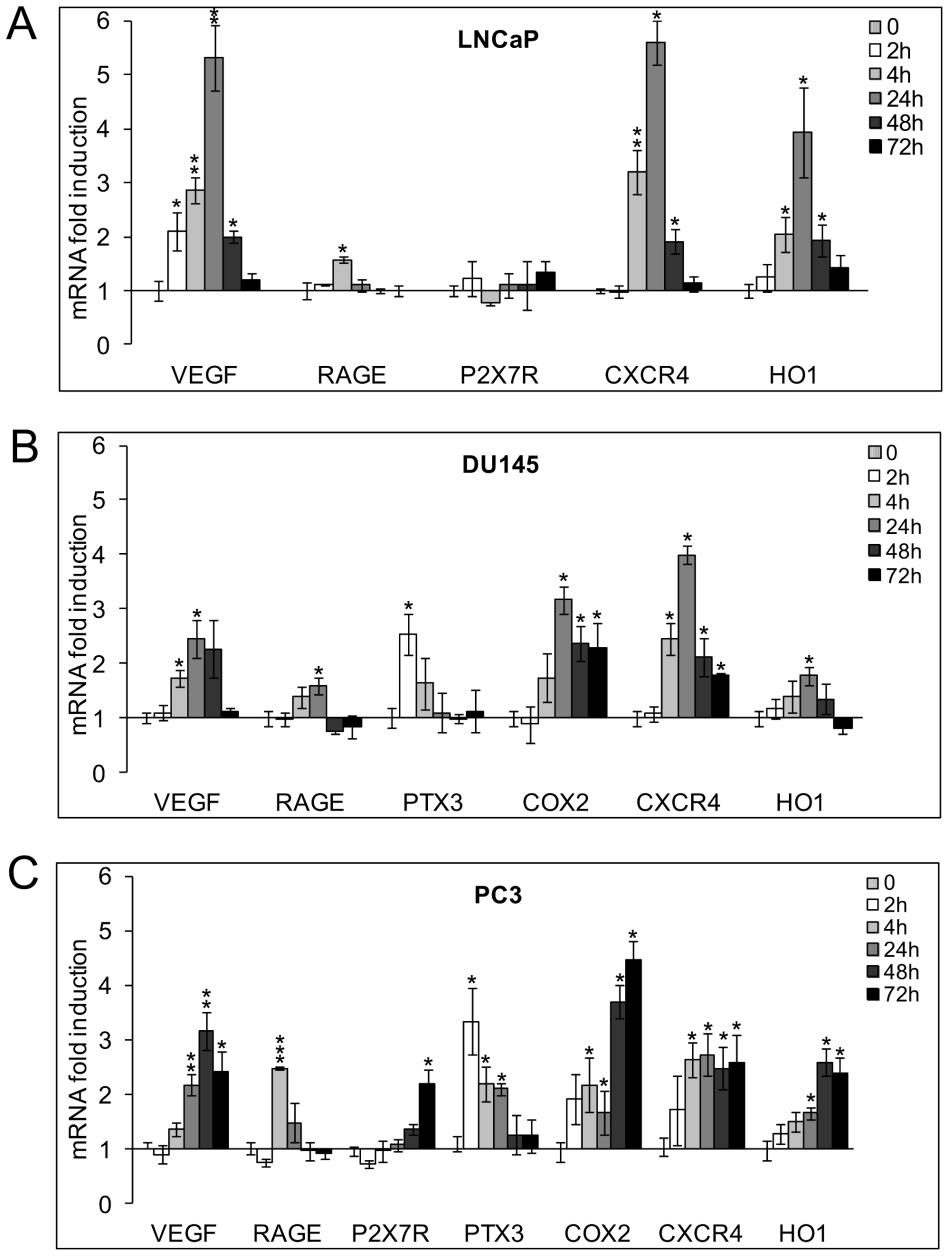
mRNA expression levels of pro-inflammatory genes in hypoxic LNCaP, DU145 and PC3 cells. Cells were exposed to 1% O_2_ from 2 up to 72 h. mRNA levels for VEGF, RAGE, P2X7R, PTX3, COX2, CXCR4 and HO1 were analyzed by real-time PCR and normalized to the housekeeping gene 18S rRNA. Graphs show the ratio between the expression of hypoxia treated cells with respect to normoxic control cells (set at 1). Mean values ± SE. * Hypoxic cells *vs* normoxic control cells.

Hypoxia was able to induce VEGF, RAGE, CXCR4 and HO1 but not P2X7R mRNA transcription. RAGE mRNA increase was limited to acute stimulation (4 h), while mRNA transcription of the other genes reached a peak after 24 h oxygen deprivation and declined by 72 h (Figure 3A).

DU145 and PC3 cells expressed a higher number of the selected inflammatory-related genes that were upregulated in hypoxia with a more complete and persistent response in more aggressive PC3 cells. In particular, DU145 evidenced basal transcripts for all the studied molecules except for P2X7R. In hypoxia, PTX3 mRNA showed a precocious and time-limited increase, while the transcription of VEGF, RAGE, COX2, CXCR4 and HO1 peaked after 24 h stimulation and declined by 72 h (Figure 3B). PC3 cells expressed detectable basal mRNA levels of all the analyzed pro-inflammatory molecules. Also in this cell line PTX3 and RAGE were maximally induced by acute hypoxia after 2 and 4 h stimulation, respectively. Different to the other cell models, the hypoxic increase of VEGF, P2X7R, COX2, CXCR4 and HO1 expression was still at its peak after 48–72 h of oxygen deprivation (Figure 3C).

Collectively, the above results highlight that the expression and hypoxic upregulation of molecules typically involved in inflammation and metastasis in prostate tumor can greatly vary according to cell phenotype.

### NF-kB inhibitor parthenolide counteracts the hypoxia induced pro-inflammatory phenotype in DU145 and in PC3 but not in LNCaP cells and induces HO1 transcription in all the cell models

**Figure 4:**
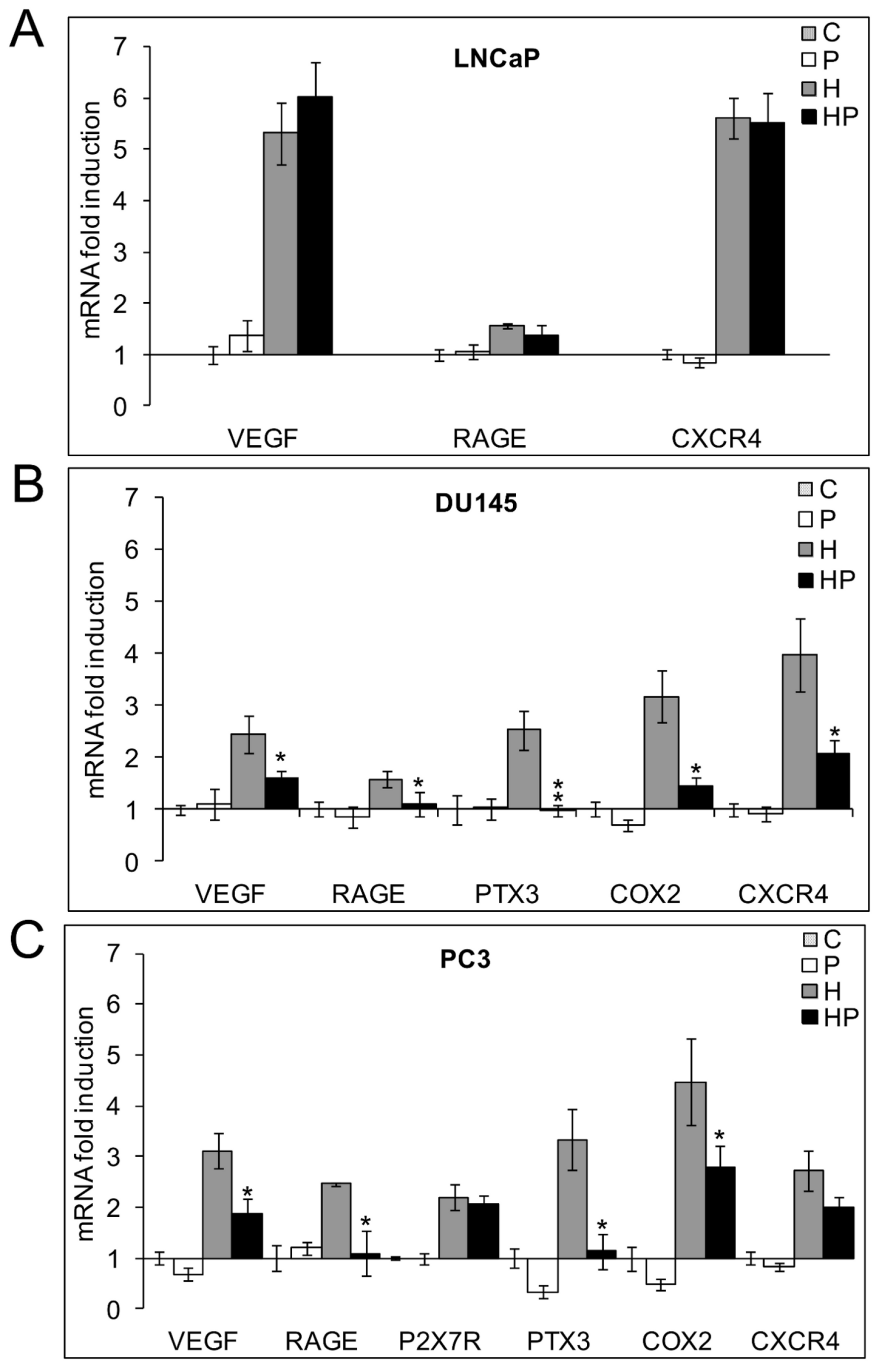
Effects of parthenolide on the expression of pro-inflammatory genes. LNCaP (**A**), PC3 (**B**), DU145 (**C**) cells were kept in normoxia and exposed to 1% O_2_ in the presence and absence of 5 µM parthenolide. mRNA levels for VEGF, RAGE, P2X7R, PTX3, COX2, CXCR4 and HO1 were analyzed by real-time PCR, normalized to the housekeeping gene 18S rRNA and expressed as fold induction with respect to the normoxic untreated control (set at 1). For each gene we present the effect of parthenolide at the time points where hypoxia exerted a maximum increase on its transcription. *Hypoxic parthenolide treated cells *vs* hypoxic cells. C: control normoxic untreated cells. P: normoxic parthenolide treated cells. H: hypoxic cells. HP: parthenolide treated hypoxic cells.

The observation that hypoxia has a different impact on NF-kB activation according to the cell line phenotype prompted us to investigate the role of NF-kB in the hypoxic upregulation of the inflammatory-related phenotype in all the prostate cells by using the natural NF-kB inhibitor parthenolide. In Figures 4A, 4B and 4C the effects of 5 µM parthenolide on the expression of the hypoxia modulated pro-inflammatory genes in LNCaP, DU145 and PC3 cells respectively are depicted, both in hypoxic and normoxic conditions. For each gene, we present the data obtained at the time point at which hypoxia exerted its maximum increase in transcription as shown in Figures 3A, 3B and 3C. Parthenolide significantly counteracted the hypoxia induced upregulation of most of these genes in DU145 and in PC3 cells except for P2X7R and CXCR4 in PC3 cells. On the contrary, the drug did not show any significant effect on the expression of the hypoxia induced molecules in less aggressive LNCaP cells.

**Figure 5:**
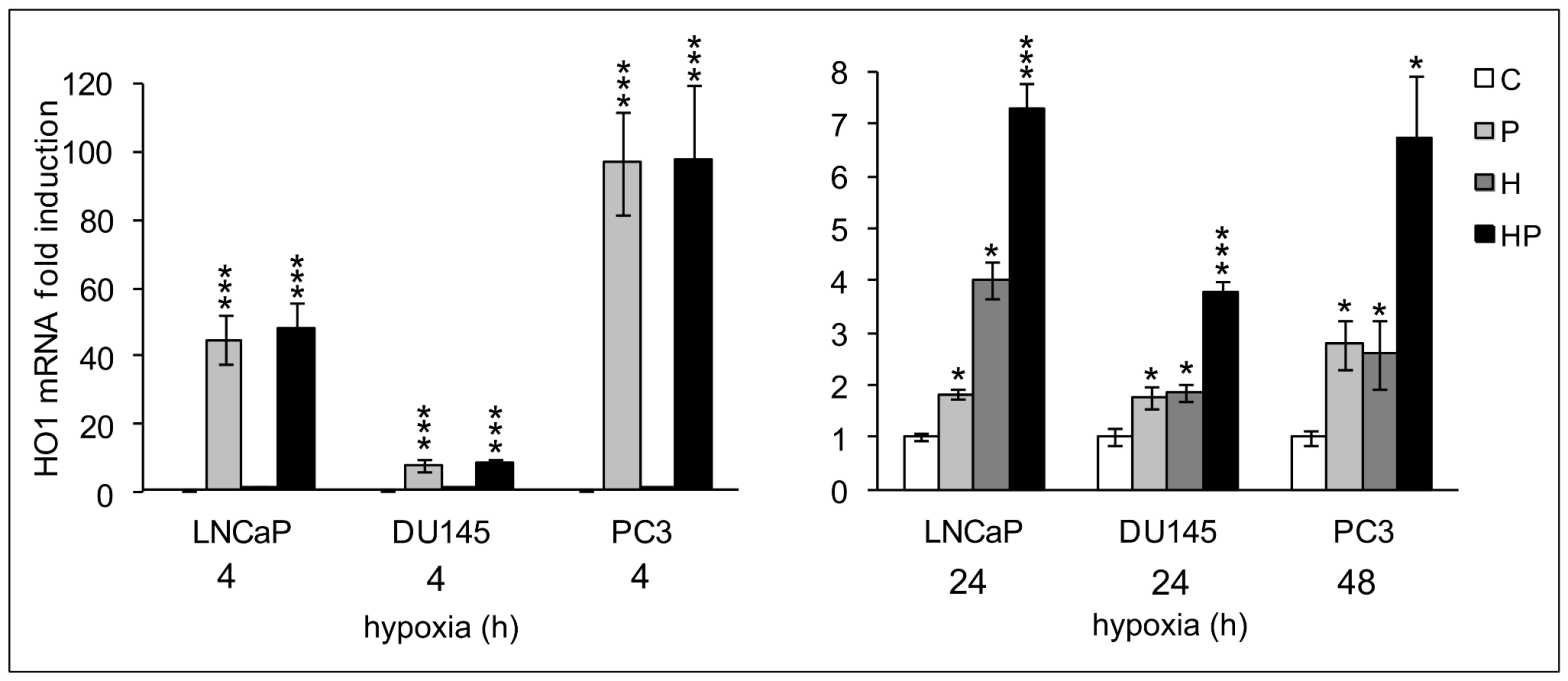
Effects of parthenolide on the expression of the anti-inflammatory gene HO1. LNCaP, PC3 and DU145 cells were kept in normoxia and exposed to 1% O_2_ in the presence and absence of 5 µM parthenolide. mRNA levels for HO1 were analyzed by real-time PCR, normalized to the housekeeping gene 18S rRNA and expressed as fold induction with respect to the normoxic untreated control (set at 1). We show the effect of parthenolide after 4 h treatment when the drug maximally upregulated HO1 expression in normoxic cultures and after 24 h (LNCaP, DU145) and 48 h (PC3) stimulation when hypoxia exerted a maximum increase on its transcription. Mean values ± SE. * Normoxic and hypoxic parthenolide treated cells *vs* normoxic control cells. C: control normoxic untreated cells. P: normoxic parthenolide treated cells. H: hypoxic cells. HP: parthenolide treated hypoxic cells.

The action of parthenolide in the modulation of HO1, a key enzyme in counteracting inflammatory damage, was completely different (Figure 5). Parthenolide was able to precociously and strongly induce HO1 in all normoxic and hypoxic prostate tumor cells with a maximal effect after 4 h stimulation. This effect gradually decreased but was still present after 24 h treatment in LNCaP and DU145 and after 48 h in PC3 cells. At these time points the effect of parthenolide and hypoxia on HO1 expression were additive.

These results demonstrate that NF-kB plays a pivotal role in shifting the more aggressive DU145 and PC3 but not LNCaP hypoxic prostate tumor cells towards a pro-inflammatory, more malignant phenotype. Furthermore, in all the cell lines, NF-kB appears to be involved in a hypoxia-independent inhibition of the anti-inflammatory gene HO1.

### Parthenolide affects HIF1α and HIF3α hypoxia dependent activation in DU145 and PC3 cell lines

**Figure 6:**
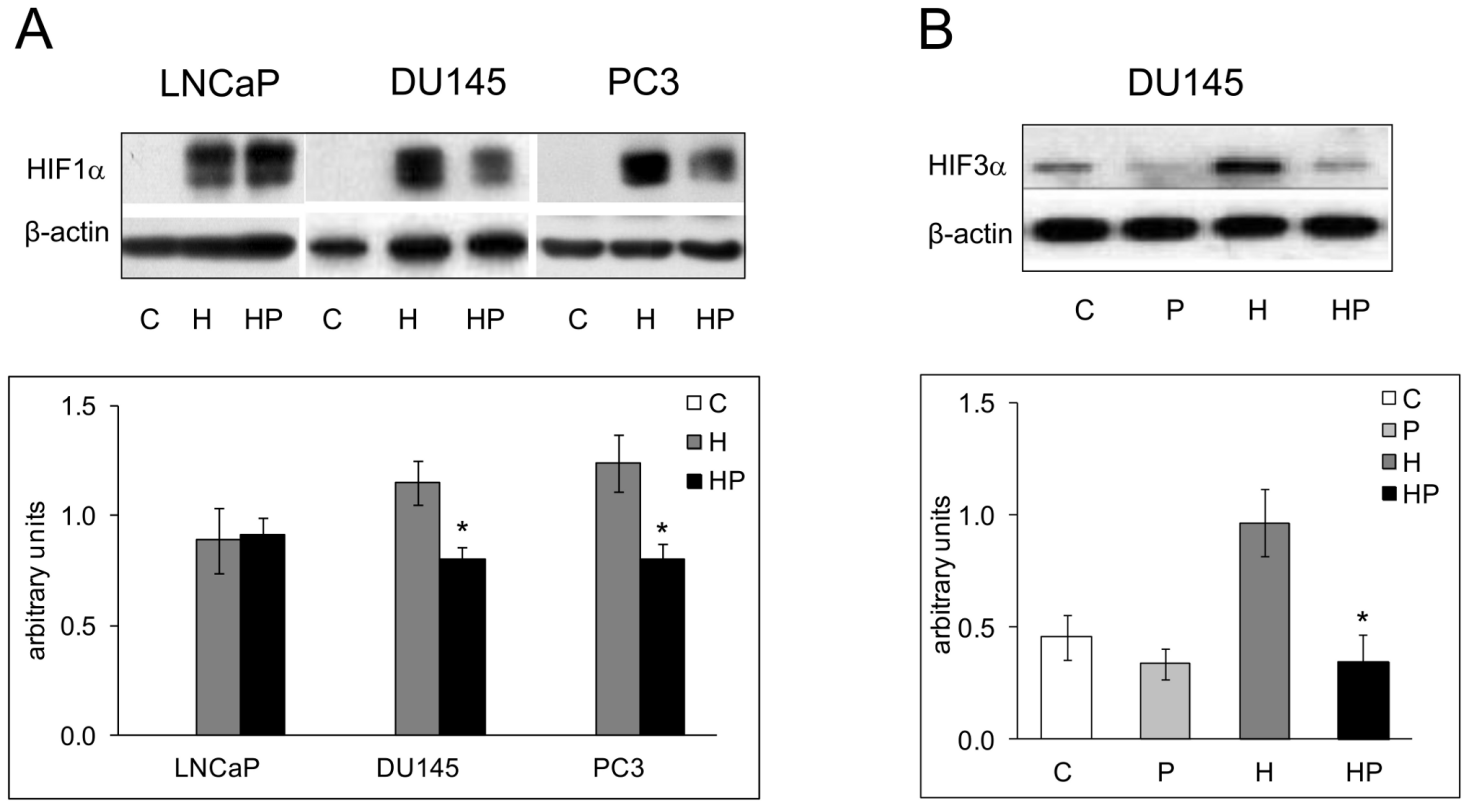
Effects of Parthenolide on HIF1α and HIF3α hypoxia dependent activation. A LNCaP, DU145 and PC3 cells were kept in normoxia and exposed to 1% O_2_ for 4 h in the presence and absence of 5 µM parthenolide. HIF1α content was measured in the nuclear fraction by immunoblot analysis. β-actin was used as a loading control. For each gene a representative experiment is shown. Mean densitometry of HIF1α relative to β-actin is also evidenced and expressed as arbitrary units. **B** DU145 cells were incubated under normoxic or hypoxic conditions for 48 h in the presence and absence of 5 µM parthenolide. HIF3α content was measured in the nuclear fraction by immunoblot analysis. β-actin was used as a loading control. For each gene a representative experiment is shown. Mean densitometry of HIF3α relative to β-actin is also evidenced and expressed as arbitrary units. Mean values ± SE. C: control normoxic untreated cells. P: normoxic parthenolide treated cells. H: hypoxic cells. HP: parthenolide treated hypoxic cells. *Hypoxic parthenolide treated cells *vs* hypoxic cells.

The control of HIFs and especially of HIF1α activation by NF-kB is a matter of debate. We tested whether parthenolide had any impact on the nuclear accumulation of HIF1α after 4 h hypoxic stimulation that induced the highest level of nuclear translocation in our experimental conditions. Figure 6A shows that the NF-kB inhibitor parthenolide significantly decreased HIF1α protein nuclear accumulation in DU145 and PC3 cell (∼30%, P<0.05). Interestingly, HIF1α nuclear level was not affected by NF-kB inhibition only in LNCaP cells that did not show a significant NF-kB activation in hypoxia.

As described in Figure 1A, HIF3α regulation was cell specific and its expression was limited to DU145 cells among the prostate tumor models examined. We investigated whether NF-kB played a role also in the hypoxia-dependent activation of HIF3α and we observed that parthenolide significantly decreased HIF3α nuclear level after 48 h hypoxic stimulation (∼65%, P<0.01) (Figure 6B).

Altogether, these data highlight that parthenolide can affect hypoxic HIF1α activation only in the tumor cells where NF-kB is responsive to oxygen deprivation. An inhibitory effect of parthenolide is observed also on HIF3α.

### Role of HIF1α in the remodeling of the pro-inflammatory phenotype in DU145 cells. Combined action of HIF1α knockdown and parthenolide

**Figure 7:**
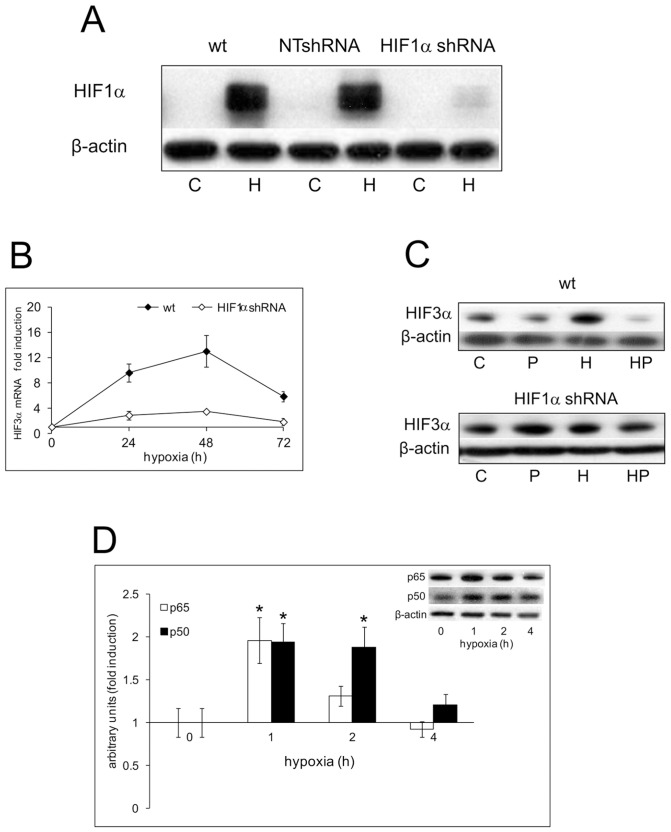
Effects of HIF1α knockdown on the hypoxic activation of HIF1α, HIF3α and of NF-kB in DU145 cells. A Wild type, NTshRNA and HIF1α shRNA transfected DU145 cells were kept in normoxia and under hypoxia (1% O_2_) for 4 h. HIF1α content was measured in the nuclear fraction by immunoblot analysis. β-actin was used as a loading control. C: control normoxic untreated cells. H: hypoxic cells. **B** Wild type and HIF1α shRNA cells were kept in normoxia and under hypoxia for 24, 48 and 72 h. mRNA level for HIF3α was analyzed by real-time PCR and normalized to the housekeeping gene 18S rRNA. The graph shows the ratio between the expression in hypoxia treated cells with respect to normoxic control cell cultures (set at 1). Mean values ± SE. **C** Wild type and HIF1α shRNA cells were kept in normoxia and under hypoxia for 48 h in the presence and absence of 5 µM parthenolide. The content of HIF3α nuclear protein was detected by immunoblot analysis. β-actin was used as a loading control. C: control normoxic untreated cells. P: normoxic parthenolide treated cells. H: hypoxic cells. HP: parthenolide treated hypoxic cells. **D** HIF1α shRNA cells were kept in normoxia and under hypoxia for 1, 2 and 4 h. The nuclear content of p50 and p65 was detected by immunoblot analysis. β-actin was used as a loading control. Densitometric analysis of p50 and p65 relative to β-actin was expressed as arbitrary units and shown as fold induction with respect to normoxic control cell cultures (set at 1). Mean values ± SE. * Hypoxic HIF1α shRNA cells *vs* HIF1α shRNA normoxic control.

Differently from LNCaP, in less differentiated DU145 and PC3 cells, NF-kB pathway was deeply involved in the remodeling of the pro-inflammatory phenotype under hypoxia. To define the contribution of HIF1α in this process, we created stable HIF1α knockdown clones (HIF1α shRNA) from DU145 cells. Figure 7A shows the level of nuclear HIF1α protein in wt, in NTshRNA vector transfected cells and in a representative HIF1α shRNA transfected clone out of the three chosen for the experiments, after 4 h oxygen deprivation. The very low level of nuclear protein in HIF1α knockdown cells derives from 87% ±1 mean drop of HIF1α mRNA (data not shown). As for wild type cells, we characterized NTshRNA and HIF1α shRNA clones in normoxia and hypoxia for the mRNA and nuclear protein level of HIF2α and HIF3α and for the NF-kB status. HIF1α knockdown cells confirmed HIF2α unresponsiveness to hypoxic treatment in our experimental conditions (data not shown). Both HIF3α mRNA and protein were induced by sustained hypoxia to a much lesser extent than in wt cells (Figures 7B, 7C). Moreover, parthenolide did not significantly affect HIF3α nuclear protein level after 48 h hypoxic stimulation (Figure 7C). Also in HIF1α knockdown cells hypoxia enhanced the activity of NF-kB as shown by the increased nuclear level of p50 and p65 in 1% oxygen (Figure 7D), even if the activation time was shorter than in wt cells.

**Figure 8:**
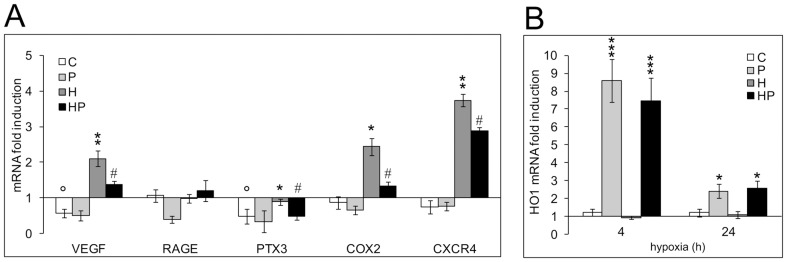
Effects of HIF1α knockdown and of parthenolide on the normoxic and hypoxic expression of the pro-inflammatory phenotype and HO1 transcription in DU145 cells. **A** HIF1α shRNA cells were exposed to 1% O_2_ or left untreated from 2 up to 72 h in the presence and absence of 5 µM parthenolide. mRNA levels for VEGF, RAGE, PTX3, COX2 and CXCR4 were analyzed by real-time PCR and normalized to the housekeeping gene 18S rRNA. For each gene we present the result on the time point where hypoxia exerted a maximum increase in transcription. Graphs show the ratio between the expression of parthenolide, hypoxia, parthenolide and hypoxia treated HIF1α shRNA cells with respect to the mean normoxic control of wt cell cultures (set at 1). Mean values ± SE. ° HIF1α shRNA normoxic control cells *vs* wt normoxic control cells, * hypoxic HIF1α shRNA cells *vs* HIF1α shRNA normoxic control cells. # Parthenolide treated hypoxic HIF1α shRNA cells *vs* hypoxic HIF1α shRNA cells. **B** Effect of parthenolide on the transcription the anti-inflammatory gene HO1 in HIF1α shRNA cells in normoxia and in hypoxia after 4 h (when parthenolide maximally upregulated HO1 expression in normoxic cultures) and 24 h treatment. Graph shows the ratio between the expression of parthenolide, hypoxia, parthenolide and hypoxia treated HIF1α shRNA cells with respect to the mean normoxic control of wt cell cultures (set at 1). Mean values ± SE. * Normoxic and hypoxic parthenolide treated HIF1α shRNA cells *vs* HIF1α shRNA normoxic control cells. C: control normoxic untreated cells. P: parthenolide treated cells. H: hypoxic cells. HP: parthenolide treated hypoxic cells.

We then measured the expression of the selected inflammatory-related genes in time-course experiments of acute (2, 4 h) and chronic (24, 48, 72 h) hypoxic stimulation in the absence and presence of 5 µM parthenolide. As expected, NTshRNA cells did not significantly differ from wt cells in all the analyzed parameters (data not shown). Therefore, we pooled the data obtained both in wt and in NTshRNA DU145 cells. Results on HIF1α shRNA cells were quite different. We compared their values with those obtained in wt cells by expressing the mRNA levels of each gene in HIF1α shRNA cells as fold induction with respect to the mean normoxic level observed in wt cells that was set at 1. Figure 8A summarizes our findings for VEGF, RAGE, PTX3, COX2 and CXCR4 gene expression at the time points where hypoxia exerted its maximum increase in transcription (2 h for PTX3, 48 h for VEGF, COX2 and CXCR4). Normoxic VEGF and PTX3 RNA values were significantly lower in knockdown compared to wt cells, confirming a role for HIF1α in their basal expression. Hypoxia significantly upregulated VEGF, COX2 and CXCR4 expression to levels comparable to those observed in wt cells. PTX3 was also induced by hypoxia but to a lesser extent with respect to wt cells. No hypoxic induction of RAGE expression was observed. Five µM parthenolide significantly counteracted the upregulation of VEGF, PTX3, COX2 and CXCR4 genes to a similar extent to what was observed in wt cells. Figure 8B depicts the effects that parthenolide, hypoxia and the combination of parthenolide and hypoxia have on HO1 expression. Different to wt cells, oxygen deprivation had no effect on HO1 transcription. However, parthenolide strongly induced HO1 expression in normoxic and hypoxic HIF1α shRNA cells after 4 h stimulation to levels comparable to those observed in wt cells. This effect gradually decreased but was still present after 24 h treatment.

These results indicate that in DU145 cells HIF1α plays a role in the normoxic expression of VEGF and PTX3 as well as in the hypoxic upregulation of PTX3, RAGE and HO1 and is highly involved in the activation of HIF3α. The hypoxic induction of VEGF, COX2 and CXCR4 is independent from HIF1α and is counteracted by parthenolide. NF-kB appears involved in a hypoxia-independent inhibition of the anti-inflammatory gene HO1.

## Discussion

The present study supports the hypothesis that the phenotype of prostate tumor epithelial cells correlates to the pathways underlying adaptation to hypoxia and to the associated pro-inflammatory gene expression occurring in the absence of CD45+ cells.

The prostate cell lines of the study derive from various metastatic prostatic carcinomas. They have been well characterized and differ in numerous features that concur to their metastatic potential and differentiation status such as androgen responsiveness, PSA expression and secretion level of growth factors and their receptors [28]. The analysis of the three cell models showed remarkable differences in the pathway bridging HIF isoforms activation and NF-kB signaling.

HIF1α was confirmed as the main player in the response to hypoxia and its changes in transcription and activation were similar in all the studied cell lines and consistent with the results already described in cells from other origin [36], [37].

On the other hand, HIF2α did not seem to play an evident role in hypoxia adaptation in the examined cell lines and, interestingly, the expression and activation of HIF3α was limited to DU145 cells. The role of HIF3α isoforms in the hypoxic induction of the pro-inflammatory transcriptoma of prostate tumor cells is still unknown. HIF3α transcription and translation in DU145 appeared strongly dependent on HIF1α. Its maximal mRNA and nuclear protein level were reached when the hypoxia-dependent increase of the pro-inflammatory genes was in decline, thus implying a complementary regulatory role in adapting to hypoxic stress. This is in agreement with the observation that HIF3α is involved in the attenuation of hypoxic pro-inflammatory response [38]. Recent reports illustrated that methylation of HIF3α could be an early event in prostate cancer development [39]. However, the observation that both LNCaP and PC3, in spite of their diverse aggressive behavior and differentiation, did not express this molecule suggested a limited role in prostate cancer progression dependent on a hypoxic microenvironment.

NF-kB is a master modulator of the pro-inflammatory response in cancer progression [40]. In our experimental conditions, we found a basal expression of p50 and p65 NF-kB subunits in the nuclei of all the considered cell lines. In DU145 and PC3 cells, hypoxia rapidly increased the level of both subunits but no detectable effect was seen in LNCaP cells, even when we assayed the formation of nuclear phospho–Ser^276^ p65 (data not shown). The high basal level of NF-kB observed in LNCaP cells could account for its reduced induction by exogenous stimuli. However, Wong et al. demonstrated that exposure to THP-1 macrophages conditioned media enabled LNCaP cells to elicit NF-kB activation and the production of local cytokines, showing that an additional effect is still possible [41]. Therefore, the NF-kB pathway seems more responsive to oxygen deprivation in cells characterized by a more aggressive and dedifferentiated tumor phenotype than in those mimicking the androgen-dependent cancer such as LNCaP. Further studies in primary and tumor cells of various differentiation statuses are needed to support this hypothesis.

Oxygen deprivation and NF-kB activation trigger the upregulation of the pro-inflammatory phenotype of prostate tumor cells, in the absence of contaminant CD45-positive cells [26]. When we analyzed the pro-inflammatory gene expression in our cell models we observed qualitative and quantitative differences in the basal and hypoxic levels of a selected set of molecules, in relation to cell phenotype. LNCaP expressed a limited number and low levels of the selected genes and only part of them were significantly induced under hypoxia, while hormone-independent cells had higher normoxic expression of almost all genes that were upregulated by hypoxia, with a more complete and persistent response in the scarcely differentiated PC3 cells. Therefore, again, LNCaP cells resulted less reactive to oxygen deprivation with respect to the other cell lines.

We used the NF-kB inhibitor parthenolide [42]–[44], to counteract the hypoxia induced pro-inflammatory gene expression. Treatment of hypoxic cells with this molecule pointed out that the induction of the pro-inflammatory phenotype could be efficiently counteracted by interfering with NF-kB pathway only in DU145 and PC3 cells where parthenolide partially impaired also hypoxic HIF1α and, when expressed, HIF3α activation. All these observations lead us to hypothesize that in androgen-independent tumors the contribution of epithelial cells to pro-inflammatory gene expression in both normoxia and hypoxia could be greater compared to less advanced cancers.

In addition, they suggest that NF-kB could play a crucial role in shifting some hypoxic tumor prostate phenotypes (namely hormone-independent cells) but not others, towards a pro-inflammatory more malignant progression. This assumption should be confirmed in additional cell models.

On the other hand, the inhibitory action of NF-kB on the expression of the anti-inflammatory gene HO1 was independent from oxygen deprivation and from tumor cell phenotype providing a rationale for interfering with the NF-kB pathway in a wide range of prostate cancers. The observation that parthenolide affected also HIF1α activation in DU145 and PC3 cells prompted us to dissect the relative impact of HIF1α and NF-kB in the pro-inflammatory response under hypoxia. We created DU145 cells with stable knockdown for HIF1α mRNA and compared the effects of parthenolide in both control wt cells and knockdown HIF1α shRNA under normoxic and hypoxic conditions. Silencing HIF1α did not significantly affect NF-kB activation under hypoxia. However, HIF1α was essential in the hypoxic increase of RAGE and HO1 transcription and highly involved in the activation of HIF3α. RAGE is a membrane receptor bridging inflammation and cancer [45] and is required to maintain invasion of tumor cells in the presence of hypoxic stress [46]. Conversely, HO1 counteracts NF-kB activation and shows antitumor and anti-inflammatory properties in prostate [34], [35]. Therefore, silencing HIF1α abolished both the pro-inflammatory loop activated by RAGE and abrogated the late anti-inflammatory induction of HO1 under hypoxia. However, HIF1α silencing did not suppress the hypoxic induction of VEGF, COX2, CXCR4 and PTX3 that was efficiently counterbalanced by parthenolide to levels comparable to those observed in wt cells. These results highlight the crucial role played by NF-kB in the hypoxic pro-inflammatory remodeling of HIF1α shRNA cells, suggesting that HIF1α silencing alone does not represent an attractive strategy in blocking the amplification of the pro-inflammatory gene expression induced by hypoxia in androgen-independent prostate tumor.

It is worth noting that the transcription of HO1 was maximally induced in the presence of parthenolide after 4 h stimulation in all normoxic and hypoxic prostate tumor cells, both in wt and in HIF1α knockdown cells, whereas in parthenolide untreated cells HO1 appeared stimulated under chronic hypoxia only when HIF1α signaling was intact. Therefore, this anti-inflammatory molecule seems to undergo a dual and complex regulation that needs further study, involving NF-kB as a repressor under normoxia and hypoxia and HIF1α as an inducer under hypoxia.

We can summarize our results as follows:

1. Less differentiated androgen-independent tumor prostate cancer cells DU145 and PC3 showed a higher basal normoxic expression and a more complete hypoxic induction of a representative set of pro-inflammatory genes as compared to androgen-dependent well differentiated LNCaP cells.
2. Both HIF1α and NF-kB signaling contributed to the hypoxic pro-inflammatory phenotype. However, the relevance of the two transcription factors in this process was different according to cancer cell phenotype. NF-kB was the main player in DU145 and PC3 cells, where parthenolide treatment was able to counteract both hypoxic pro-inflammatory induction and HIF1α activation. Such effects were absent in LNCaP cells.
3. In all analyzed cell models, basal NF-kB activation appeared to be responsible for the transcriptional inhibition of the anti-inflammatory enzyme HO1. This action was counteracted by parthenolide, suggesting a rationale for interfering with the NF-kB pathway in a wide range of prostate cancers.
